# Retraction: Comparative Efficacy and Long-Term Outcomes of Beta-Blockers Alone or in Combination With Angiotensin-Converting Enzyme (ACE) Inhibitors in Chronic Heart Failure: A Systematic Review

**DOI:** 10.7759/cureus.r189

**Published:** 2025-07-16

**Authors:** Waleed Hassan, Shamima A Nila, Muneeb Ahmed, David O Okello, Muhammad Maqbool, Muath M Dabas, Maryam Nour, Safiyyah M Khan, Fazeela Ansari, Natasha Anum, Sheikh Pervaiz

**Affiliations:** 1 Internal Medicine, Shaikh Zayed Hospital, Lahore, PAK; 2 Internal Medicine, Cumilla Medical College Hospital, Cumilla, BGD; 3 Internal Medicine, Rawalpindi Medical University, Rawalpindi, PAK; 4 Internal Medicine, Ministry of Health, Lusaka, ZMB; 5 Internal Medicine, Shaheed Mohtarma Benazir Bhutto Medical University, Karachi, PAK; 6 Surgery, University of Jordan, Amman, JOR; 7 Family Medicine, John F. Kennedy University School of Medicine, Willemstad, CUW; 8 Emergency Medicine, Henry Ford Health System, Detroit, USA; 9 Research Methodology, California Institute of Behavioral Neurosciences and Psychology (CIBNP), Fairfield, USA; 10 Medicine and Surgery, Dubai Medical College, Dubai, ARE; 11 Internal Medicine, Liaquat University of Medical and Health Sciences, Jamshoro, PAK; 12 Internal Medicine, Nishtar Medical University, Multan, PAK

The Editors-in-Chief have retracted this article following an ethics complaint and internal investigation. This article was linked to an authorship-for-sale scheme, with evidence showing the article was advertised in a group offering paid authorship. Several listed authors admitted to having no role in the research or writing, confirming the use of unethical authorship practices. As a result, the credibility of the article is compromised and retraction is required.

